# Imaging features of desmoplakin arrhythmogenic cardiomyopathy: A comparative cardiovascular magnetic resonance study

**DOI:** 10.1016/j.jocmr.2025.101867

**Published:** 2025-02-26

**Authors:** Mikael Laredo, Etienne Charpentier, Shannon Soulez, Vincent Nguyen, Annamaria Martino, Leonardo Calò, Flavie Ader, Alexis Hermida, Véronique Fressart, Philippe Charron, Nadjia Kachenoura, Estelle Gandjbakhch, Alban Redheuil

**Affiliations:** a Sorbonne Université, CNRS, INSERM, Laboratoire d′Imagerie Biomédicale, LIB, Paris, France; b Sorbonne Université, Département de Cardiologie, AP-HP, Hôpital Universitaire Pitié-Salpêtrière, Paris, France; c Institute of Cardiometabolism and Nutrition (IHU ICAN), Paris, France; d Sorbonne Université, Imagerie Cardiovasculaire et Thoracique (ICT), AP-HP, Hôpital Universitaire Pitié-Salpêtrière, Paris, France; e Division of Cardiology, Policlinico Casilino, Rome, Italy; f Sorbonne Université, Département de Génétique, Centre de Références des Maladies Cardiaques Héréditaires ou rares, AP-HP, Inserm UMR_1166, IHU ICAN, Hôpital Universitaire Pitié-Salpêtrière, Paris, France

**Keywords:** Arrhythmogenic cardiomyopathy, Right or left dominant, Biventricular, Cardiac magnetic resonance imaging, Myocardial fibrosis

## Abstract

**Background:**

Arrhythmogenic cardiomyopathy (ACM) related to Desmoplakin (*DSP*) mutations is a distinct condition associated with particularly severe outcomes, more frequent left ventricular (LV) involvement, including fibrosis, dysfunction, and inflammatory episodes. Whether *DSP*-ACM is associated with specific imaging features remains elusive. This study aims to provide a comprehensive description of cardiovascular magnetic resonance (CMR) findings in patients with *DSP*-ACM and to compare them to RV-dominant ACM with LV involvement (LV+ right-dominant-ACM).

**Methods:**

Patients with *DSP*-ACM matched with patients with ACM related to a non-*DSP* desmosomal mutation and ≥1 feature of LV involvement underwent CMR in two institutions. Biventricular metrics and segmental wall motion abnormalities (WMA) were assessed. LV late gadolinium enhancement (LGE) was assessed both qualitatively and quantitatively after semi-automated segmentation.

**Results:**

Overall, 70 ACM patients were analyzed; 37 with *DSP*-ACM and 33 in the LV+ right-dominant-ACM group. LVEF was significantly lower in the *DSP*-ACM group (46 ± 12%) than in the LV+ right-dominant-ACM group (56 ± 10%, P = 0.001). Conversely, RVEF was significantly higher in the *DSP*-ACM group (45 ± 11% vs. 40 ± 12%, P = 0.04) and both RV end-diastolic (100 ± 24 vs 130 ± 44 mL/m², P = 0.002) and end-systolic (56 ± 21 vs 81 ± 45 mL/m², P = 0.007) indexed volumes were significantly smaller in *DSP*-ACM as compared to the LV+ right-dominant-ACM group. The LV to RV end-systolic volume ratio (0.96 [interquartile range (IQR)0.70–1.27] vs. 0.59 [IQR 0.48—0.69]) was significantly higher in the *DSP*-ACM group (P < 0.0001), and had a good performance in differentiating both groups (area under the ROC curve 0.86, optimal threshold 0.8). Patients in the *DSP*-ACM group had significantly more LV and less RV WMA than those in the LV+ right-dominant-ACM group. The amount of LGE was significantly higher in the *DSP* group (14% ± 16 vs. 2%±3, P < 0.0001) and present in the majority of LV segments, particularly in the lateral and inferior walls, as compared to LV+ right-dominant-ACM patients. Transmural LGE and the presence of a ring-like pattern corresponding to circumferential subepicardial LGE involving ≥3 contiguous LV basal segments were highly specific of *DSP*-ACM.

**Conclusion:**

The presence of LV to RV end-systolic volume ratio>0.8, global LGE>5%, transmural and/or a ring-like LGE pattern are highly suggestive of DSP-ACM and should prompt careful diagnostic assessment considering the severe associated outcome.

## Graphical abstract

**Figure:**
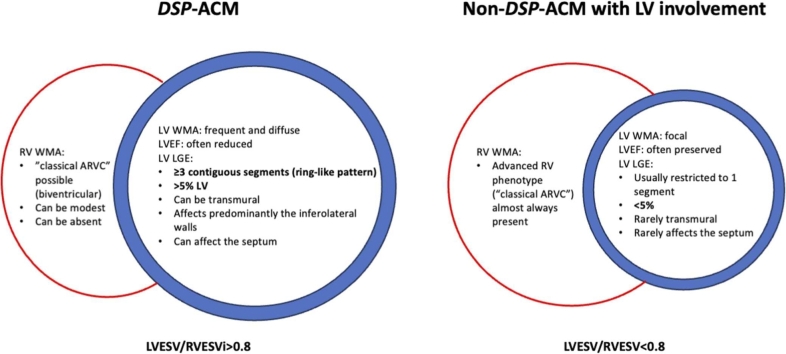

## 1 Introduction

Arrhythmogenic cardiomyopathy (ACM) is a heterogeneous genetic disease characterized by fibrofatty infiltration of the myocardium and the development of potentially lethal ventricular arrhythmias (VA) or heart failure (HF) [1]. Desmoplakin (*DSP*) is a crucial cardiac desmosomal protein responsible for transmitting myocardial force and with pathogenic variants in the *DSP* gene being significant contributors to ACMs [2], [3]. Although historically linked to the development of arrhythmogenic right ventricular cardiomyopathy (ARVC), *DSP*-related ACM has unique characteristics and is increasingly recognized as a distinct clinical condition [4], [5], [6], [7]. Patients with *DSP*-related ACM exhibit a high frequency of left ventricular (LV) abnormalities, including myocardial fibrosis and systolic regional and global dysfunction [4], [5], [8], [9]. They may also experience episodes of inflammatory myocardial damage and face an increased risk of life-threatening VA [5]. While risk stratification of patients with predominant right ventricle (RV)-involvement ARVC has significantly improved in the last decade, management of *DSP*-ACM patients remains difficult and probably requires specific consideration. The recognition of a *DSP*-ACM phenotype parallels the nosological evolution of the ACM landscape and the increasing individualization of arrhythmogenic left ventricular cardiomyopathy (ALVC) wherein the LV is predominantly affected, even at early stages [7]. However, our ability to comprehensively characterize a *DSP*-ACM phenotype in particular is hindered by relatively small cohorts often confounded by other ALVC-related genotypes.

Cardiovascular magnetic resonance (CMR) imaging, due to its ability to characterize both functional and structural ventricular abnormalities, has become a cornerstone of ACM diagnosis and classification [7], [10]. While CMR features of ARVC have been established since the 2010 Task Force Criteria (TFC), the CMR characterization of ALVC has been more recently reported in patients including various desmosomal and non-desmosomal pathogenic variants [11], [12], [13], [14]. These features typically include global or regional LV dysfunction associated with a variable degree of RV global or regional dysfunction and diffuse LV late gadolinium enhancement (LGE) with a subepicardial pattern [7], [15], [16]. The latter, a CMR subepicardial fibrosis surrogate, is increasingly considered a typical hallmark of ALVC in comparison to dilated cardiomyopathy (DCM) more prone to display intramural LGE.

LV structural alterations can also be seen in “classic” ARVC, especially at more advanced disease stages, questioning whether they are specific of ALVC in general and *DSP*-ACM in particular. Moreover, because *DSP*-ACM is associated with a particularly severe outcome and requires specific risk stratification tools, the characterization of CMR *DSP*-ACM features is important for early disease recognition, especially in atypical presentations such as acute myocarditis.

To address this conundrum, we hereby provide a comprehensive CMR description of biventricular involvement in *DSP*-ACM and a comparison with non-*DSP* desmosomal ACM with LV involvement.

## 2 Methods

### 2.1 Study design

We performed a retrospective observational study in two European tertiary institutions for ACM diagnosis and management: Sorbonne Université, AP-HP, Groupe Hospitalier Pitié-Salpêtrière, Paris, France and Policlinico Casilino, Rome, Italy. All patients gave written consent for the use of their routine clinical data in research, including data from genetical analyses. This study complies with the reference methodology (MR004) of the French National Commission for Informatics and Liberties.

### 2.2 Patient population

Patients with a *DSP* pathogenic or likely pathogenic (P/LP) variant [17] and a phenotypical manifestation of ACM (*DSP*-ACM) were matched by age, gender, and body surface area to patients with ACM with a non-*DSP* P/LP variant in a desmosome gene with at least one morphological feature of LV involvement (LV+ right-dominant-ACM). The *DSP*-ACM group comprised patients with a *DSP* P/LP variant who fulfilled definite 2010 ARVC TFC [18] or at least one 2020 International Consensus (IC) criterion for ALVC besides the family history/genetics criterion [7]. Family members carrying a *DSP* P/LP variant who did not exhibit any phenotypical feature of ACM were not included. The LV+ right-dominant-ACM group comprised patients with (1) a P/LP variant in a desmosomal gene of the ARVC spectrum (*PKP2*, *DSG2*, *DSC2*, *JUP*) and who (2) fulfilled definite 2010 ARVC TFC [18] or at least one 2020 IC criterion for ALVC [7] besides the family history/genetics criterion and (3) had at least one feature of morphological LV involvement among the following: LV global dysfunction, LV wall motion abnormality (WMA), or non-ischemic LV LGE. Patients with genotype negative ACM and patients with a P/LP variant in a non-desmosomal gene associated with ALVC (such as *FLNC*, PLN, *TMEM43, LMNA*) were not included.

### 2.3 ARVC/ALVC criteria

Clinical data used for 2010 TFC ARVC and 2020 IC ALVC definitions were retrieved from the patients’ medical file at the time of CMR evaluation. CMR analyses were solely used to evaluate the fulfillment of morphological criteria for both 2010 TFC ARVC and 2020 IC ALVC criteria.

For the characterization of ARVC-type ACM, the 2010 ARVC TFC criteria were used. The 2020 IC ARVC criteria were not used because only a minority of CMR exams included high-resolution three-dimensional (3D) RV LGE evaluation, hampering the use of 2020 IC criteria for the characterization of the study population. Consequently, static RV morphological abnormalities, defined below, were not considered as a diagnostic criterion. Per definition of our study population, all patients met the major family history/genetics criterion.

For the characterization of ALVC-type ACM, the 2020 IC ALVC criteria were used [7]. Global LV dysfunction was defined as left ventricular ejection fraction (LVEF) <57%, in accordance with the 2020 IC ALVC criteria, no patient in the study population was a professional athlete. Regional LV WMA was defined as regional LV hypokinesia or akinesia of the LV free wall, septum, or both. LV structural myocardial abnormalities were defined as ≥1 LV segment harboring positive LGE, excluding septal junctional LGE.

### 2.4 Cardiovascular magnetic resonance

#### 2.4.1 CMR acquisition

CMR examinations were performed according to standard clinical procedures using a 1.5T system (Magnetom Aera, Siemens Healthineers, Erlangen, Germany) for all patients in a department dedicated to cardiovascular imaging. CMR required electrocardiogram (ECG) gating and an 18-element thoracic surface coil. Cine acquisitions were performed using an steady-state free precession (SSFP) sequence in LV long-axis two-, three-, four-chamber, short-axis, and RV-specific views including multiple RV two-chamber views and two orthogonal RV outflow tract views stacks, using the following typical parameters: acquisition matrix = 256 × 256, repetition time = 51 ms, echo time = 1.2 ms, flip angle = 53°, pixel size = 1.48 mm × 1.48 mm, slice thickness = 6 mm, inter-slice gap = 1 mm, 40 temporal cardiac phases with adjustment of the number of views per segment according to the heart rate to achieve high temporal resolution. Regarding tissue characterization acquisitions: (1) short- and long-axis radial LGE T1-weighted inversion-recovery images were acquired 10 min after intravenous (IV) injection of 0.2 mmol/kg of Gd-DTPA (Dotarem, Guerbet, Villepinte, France) with inversion time chosen to null the normal myocardium, (2) motion-corrected basal, and mid-LV short-axis modified look-locker inversion-recovery T1 mapping images were acquired before and 15 min after contrast injection, (3) motion-corrected basal and mid-LV short-axis T2 mapping imaging using a three-point T2-prepared SSFP sequence was performed before contrast injection.

#### 2.4.2 Volumetric and feature-tracking strain analyses

The LV and RV end-diastolic and end-systolic volumes, LV mass, LVEF, and RVEF were estimated following a semi-automated tracing of the myocardial contours on successive cine SSFP short-axis slices. Biventricular volumetric and LV feature-tracking analyses were performed using cvi42 (Circle Cardiovascular Imaging, Calgary, Alberta, Canada) by a single operator (E.C.) blinded to clinical, genetic, and visual WMA data.

#### 2.4.3 Dynamic and static biventricular morphological abnormalities

Regional biventricular WMAs were assessed independently by two cardiovascular radiologists expert in ACM diagnosis (A.R. and E.C., with 20 and 5 years’ experience, respectively) blinded to genetic and clinical data. In case of discrepancy, the images were openly discussed by the two operators and a consensus reached. The LV was segmented using the 17 American Heart Association (AHA) segments [19]. The RV was divided into 10 segments, according to an in-house RV segmentation providing a comprehensive coverage of the RV [1].

WMAs included hypokinesia, akinesia, and dyskinesia for the LV and hypokinesia, akinesia, and dyskinesia/dyssynchrony for the RV. Static RV morphological abnormalities included *scalloping*, defined as small localized akinetic or dyskinetic bulges with notable wall thinning often seen at the infundibulum or sub-infundibulum and peri-tricuspid basal RV inferior or inferior-free wall angle and with a “cauliflower” aspect when multiple and surrounded by trabeculated myocardium in the free wall and apical RV regions; *bulging*, defined as a localized outward protrusion throughout the cardiac cycle; and *aneurysms*, defined as a larger akinetic or dyskinetic bulge involving a significant portion or entire RV segment.

#### 2.4.4 Analyses of late gadolinium enhancement and mapping parameters

For each patient, LV endocardial and epicardial contours were drawn on all LGE images by an experienced radiologist (E.C.) using cvi42.

The presence of LV LGE was assessed visually for each of the 17 LV segments. The LGE pattern was classified as subepicardial, intramyocardial, subendocardial, or transmural, the latter being defined by radial LGE extension >80% of segmental thickness. Regional LGE was visually scored using a binary scale of <50% or ≥50% of radial segmental extent. Specific junctional LGE spots at the LV insertion points of the RV were not considered. A ring pattern was defined as the presence within a short-axis slice of ≥3 contiguous basal LV segments (≥50% of the LV circumference) with subepicardial LGE, irrespective of radial extent [20]. For LGE quantification, LGE was automatically identified within the segmented myocardium using unsupervised clustering based on the fuzzy c-mean [21], [22] and the percentage of LGE was calculated as the ratio between the total amount of LGE divided by the total myocardial volume. Global native T1 and T2 times were calculated by averaging relaxation times over the basal and mid-LV slices. Myocardial segmentation was performed semi-automatically and with manual correction and validation by an experienced radiologist (E.C.) using cvi42. Care was taken to avoid pixels belonging either to the ventricular cavities or epicardial fat.

### 2.5 Patients’ follow-up

Patients were followed-up in the two above-mentioned academic institutions. Death, heart transplantation, sustained VA, and HF events were collected. We defined a composite outcome, including the occurrence of VA or HF or heart transplantation.

### 2.6 Statistical analyses

Data are presented as mean ± standard deviation (SD) or median (interquartile range [IQR]), depending on the variables’ distribution assessed by the Shapiro-Wilk test. Statistical tests involved the Pearson chi-square or the Fisher exact test for comparing categorical variables and Student t-test or Wilcoxon test for continuous variables, depending on the variables’ distribution. The association between two continuous variables was evaluated using Pearson's correlation coefficient *r*; when one or more assumptions of normality were refuted, Spearman's rank correlation coefficient ρ was used instead. For variables of interest, receiver operating characteristic (ROC) curves were built, and the area under the ROC curve (AUC) was calculated. The Youden index was used to determine the optimal threshold value in terms of sensitivity/specificity. Logistic regression analyses were used to assess the association between clinical outcomes and imaging parameters. All statistical tests were two-sided and were considered significant at P < 0.05. All statistical analyses were performed using JMP Pro v15.2.0 (SAS Institute Inc., Cary, North Carolina). Data are available upon reasonable request.

## 3 Results

### 3.1 Study population

Overall, 70 ACM patients who underwent CMR examination in a period ranging from January 2014 to September 2022 and met inclusion criteria were retrospectively included. The *DSP* group comprised 37 patients while the LV+ right-dominant-ACM group comprised 33 patients.

### 3.2 Clinical characteristics

In the LV+ right-dominant-ACM group, 23/33 (70%) patients harbored a P/LP variant in *PKP2*, 8/33 (24%) patients in *DSG2*; 2/33 (6%) patients harbored a dual *PKP2*-*DSG2* genotype with both variants being classified as P/LP. Among clinical characteristics at the time of CMR evaluation (Table 1), there was no difference in proband status or clinical presentation in both groups, except for history of resuscitated sudden cardiac death (4/37 [11%] patients vs none, P = 0.02) and myocarditis (11/37 [30%] vs 3/33 [9%] patients, P = 0.03), which were significantly more frequent in the *DSP* group than in the LV+ right-dominant-ACM group.

**Table 1:** Clinical characteristics of patients with *DSP* and LV+ right-dominant arrhythmogenic cardiomyopathy.

	All patients (n = 70)	*DSP-ACM* (n = 37)	LV+ right-dominant ACM (n = 33)	P value
Male sex	46 (66)	24 (65)	22 (67)	0.87
Age at CMR evaluation, years	44±16	45±16	43±17	0.58
Body surface area, kg cm^−2^	1.9 (1.7–2.0)	1.8 (1.7–2.0)	1.9 (1.8–2.0)	0.10
Class IV/V variants in the ACM spectrum	70 (100)			
*DSP*	-	37 (100)	-	
*PKP2*	-	-	23 (70)	
*DSG2*	-	-	8 (24)	
*PKP2+DSG2*	-	-	2 (6)	
Proband	46 (66)	25 (56)	21 (63)	0.73
Clinical presentation				
Sustained VA	17 (25)	10 (25)	7 (21)	0.57
Non-sustained VA	26 (37)	13 (35)	20 (61)	0.71
Sudden cardiac death	4 (6)	4 (11)	0	0.02
Myocarditis	14 (20)	11 (30)	3 (9)	0.03
Non-ischemic chest pain	15 (21)	12 (32)	3 (9)	0.01
Heart failure	5 (7)	4 (11)	1 (3)	0.19
Syncope	8 (11)	4 (11)	4 (12)	0.86
24 h Holter-ECG PVC count	650 (1999–2000)	535 (191–1392)	830 (242–2000)	0.39
ICD implanted^a^	18 (26)	13 (35)	5 (15)	0.05

Data are n (%), median (interquartile range), or mean ± standard deviation

*ACM* arrhythmogenic cardiomyopathy, *CMR* cardiovascular magnetic resonance, *ECG* electrocardiogram, *ICD* implantable cardioverter defibrillator, *DSG2* desmoglein 2, *DSP* desmoplakin, *LV* left ventricular, *PKP2* plakophilin-2, *PVC* premature ventricular contraction, *SD* standard deviation, *VA* ventricular arrhythmia

a All ICD were implanted after CMR evaluation

A definite ARVC diagnosis (2010 TFC) was met significantly less frequently in the *DSP* group (23/37 [62%] patients) than in the LV+ right-dominant-ACM group (30/33 [91%] patients, P = 0.01). The TFC score (2010) was significantly lower in the *DSP* group (4 [IQR 3–5.5]) than in the LV+ right-dominant-ACM group (7 [IQR 5.5–8], P < 0.0001). 2010 TFC ARVC and 2020 IC ALVC criteria across groups are shown in Table 2. Specifically, the *DSP* group comprised fewer patients with major RV global or regional dysfunction (14/37 [38%] vs 25/33 [76%], P = 0.001), major repolarization criteria (8/37 [22%] vs 17/33 [51%], P = 0.01), and major depolarization criteria (1/37 [3%] vs 10/33 [30%], P = 0.001) than the LV+ right-dominant-ACM group. The distribution of the 2010 TFC ARVC score in each group is available in Supplemental Fig. 1. There was no significant difference in the presence of 2020 IC ALVC criteria between both groups, including morphofunctional LV abnormalities, ECG, and arrhythmia criteria (Table 2).

**Table 2:** 2010 ARVC Task Force Criteria and 2020 ALVC International Criteria in patients with *DSP* and LV+ right-dominant arrhythmogenic cardiomyopathy.

	All patients (n = 70)	*DSP-ACM* (n = 37)	LV+ right-dominant ACM (n = 33)	P value
2010 TFC ARVC criteria				
RV global or regional dysfunction				
Major	39 (56)	14 (38)	25 (76)	0.001
Minor	14 (20)	8 (22)	6 (18)	0.77
Repolarization abnormalities				
Major	25 (36)	8 (22)	17 (51)	0.01
Minor	9 (13)	3 (8)	6 (18)	0.29
Depolarization abnormalities				
Major	11 (26)	1 (3)	10 (30)	0.01
Minor	16 (23)	5 (14)	11 (33)	0.09
Ventricular arrhythmias				
Major	13 (19)	6 (16)	7 (21)	0.76
Minor	31 (44)	14 (38)	17 (58)	0.34
Family history/genetics				
Major	70 (100)	37 (100)	33 (100)	-
2010 TFC ARVC score	5 (4–7)	4 (3–6)	7 (6–8)	<0.0001
2010 TFC ARVC diagnosis				
Definite	53 (76)	23 (62)	30 (91)	0.01
Borderline	9 (13)	7 (19)	2 (6)	0.16
Possible	8 (11)	7 (19)	1 (3)	0.06
2020 International Consensus ALVC criteria				
Morphofunctional LV abnormalities				
Global LV systolic dysfunction^a^	45 (64)	24 (65)	21 (64)	1.00
Regional LV wall motion abnormality^b^	8 (11)	6 (16)	2 (6)	0.27
LV structural myocardial abnormalities^c^	57 (81)	32 (86)	25 (75)	0.12
LV repolarization abnormalities^d^	22 (31)	10 (27)	12 (36)	0.40
LV depolarization abnormalities^e^	15 (21)	8 (22)	7 (21)	1.00
Ventricular arrhythmias^f^	35 (50)	23 (62)	12 (36)	0.05

Data are n (%), median (interquartile range), or mean ± standard deviation

*ACM* arrhythmogenic cardiomyopathy, *ALVC* arrhythmogenic left ventricular cardiomyopathy, *ARVC* arrhythmogenic right ventricular cardiomyopathy, *CMR* cardiovascular magnetic resonance, *DSP* desmoplakin, *LV* left ventricle, *LVEF* left ventricular ejection fraction, *RV* right ventricle, *TFC* Task Force Criteria, *VA* ventricular arrhythmia

a Defined as LVEF <58% on cine cardiac magnetic resonance

b Regional LV hypokinesia or akinesia of LV free wall, septum, or both, by CMR, in the absence of global LV systolic dysfunction

c Defined as late gadolinium enhancement (stria pattern) of ≥1 the LV

d Defined as T-wave inversion in left precordial leads

e Defined as low QRS voltage in limb leads

f >500 premature ventricular contractions per 24 h, or non-sustained or sustained VA with right bundle branch block morphology. All these definitions according to the 2020 International Criteria for LV phenotype REF

### 3.3 Cardiac magnetic resonance findings

#### 3.3.1 Volumetric and feature-tracking parameters

The LVEF was significantly lower in the *DSP* group (46 ± 12%) than in the LV+ right-dominant-ACM group (56 ± 10%, P = 0.001) with more patients having a moderate to severe global LV systolic dysfunction defined as LVEF ≤45% in the *DSP* group than the LV+ right-dominant-ACM group (15/37 [42%] patients vs 1/33 [3%] patient, P < 0.0001). However, there was no significant difference in LV volumes (Table 3). Global LV longitudinal (−15 ± 4 vs −17 ± 4%, P = 0.02), radial (21 ± 8 vs 28 ± 8%, P = 0.0006) and circumferential (−14 ± 4 vs −17 ± 4%, P = 0.0008) peak strains were significantly lower in the *DSP* group than in the LV+ right-dominant-ACM group.

**Table 3:** Cardiac magnetic resonance findings.

	All patients (n = 70)	*DSP-ACM* (n = 37)	LV+ right-dominant ACM (n = 33)	P value
Volumetric and feature-tracking parameters				
LVEDVi, mL/m²	97±24	99±24	95±23	0.40
LVESVi, mL/m²	49±25	55±25	44±24	0.09
LVEF, %	50±12	46±12	56±10	0.001
LVEF ≤ 45%, n (%)	16 (24)	15 (42)	1 (3)	<0.0001
LV mass, g/m²	53±11	52±12	52±10	0.90
Global LV longitudinal strain, %	−16±4	−15±4	−17±4	0.02
Global LV radial strain, %	24±9	21±8	28±8	0.0006
Global LV circumferential strain, %	−15±4	−14±4	−17±4	0.0008
RVEDVi, mL/m²	116±39	100±24	130±44	0.002
RVESVi, mL/m²	69±37	56±21	81±45	0.007
RVEF, %	42±11	45±11	40±12	0.04
RVEF ≤ 40%	25 (37)	10 (28)	15 (47)	0.10
LVEDV/RVEDV	0.84 (0.75–1.01)	1.01 (0.81–1.18)	0.78 (0.68–0.85)	<0.0001
LVESV/RVESV	0.68 (0.56–0.96)	0.96 (0.70–1.27)	0.59 (0.48–0.69)	<0.0001
Morphofunctional abnormalities				
LV WMA	37 (53)	22 (59)	15 (45)	0.2
Anterior LV WMA	12 (17)	8 (22)	4 (12)	0.26
Lateral LV WMA	29 (42)	19 (53)	10 (30)	0.04
Inferior LV WMA	22 (31)	15 (42)	7 (21)	0.06
Septal LV WMA	11 (16)	8 (22)	3 (9)	0.13
RV WMA (akinesia or dyskinesia)	20 (56)	20 (56)	26 (79)	0.04
RV bulging	16 (23)	6 (16)	10 (30)	0.16
LV tissue characterization				
LGE presence	57 (83)	31 (86)	26 (79)	0.42
Anterior wall LGE	26 (38)	20 (56)	6 (18)	0.001
Lateral wall LGE	49 (71)	26 (72)	23 (70)	0.81
Inferior wall LGE	31 (55)	24 (67)	14 (42)	0.04
Septal LGE	28 (41)	22 (61)	6 (18)	0.0006
Subendocardial LGE	1 (1)	1 (1)	0	0.25
Subepicardial LGE	55 (80)	31 (86)	24 (72)	0.17
Intramyocardial LGE	10 (14)	5 (14)	5 (15)	0.88
LGE, %	9±14	14±16	2±3	<0.0001
Global native T1,^a^ ms	1035±54	1042±58	1017±39	0.18
Global native T2,^b^ ms	50±3	50±3	50±40	0.54

Data are n (%), median (interquartile range), or mean ± standard deviation

*ACM* arrhythmogenic cardiomyopathy, *DSP* desmoplakin, *LGE* late gadolinium enhancement, *LV* left ventricle, *LVEDV* left ventricular end-diastolic volume, *LVEDVi* indexed left ventricular end-diastolic volume, *LVESV* left ventricular end-systolic volume, *LVEF* left ventricular ejection fraction, *RV* right ventricle, *RVEDV* right ventricular end-diastolic volume, *RVEDVi* indexed right ventricular end-diastolic volume, *RVESV* right ventricular end-systolic volume, *RVESVi* indexed right ventricular end-systolic volume, *RVEF* right ventricular ejection fraction, *WMA* wall motion abnormalities

a Data available in 32 patients only (23 in the *DSP* group and 9 in the LV+ right-dominant-ACM group)

b Data available in 31 patients only (22 in the *DSP* group and 9 in the LV+ right-dominant-ACM group)

Conversely, the RVEF was significantly higher in the *DSP* group than in the LV+ right-dominant-ACM group (45 ± 11% vs 40 ± 12%, P = 0.04) and both indexed RV end-diastolic (100 ± 24 vs 130 ± 44 mL/m², P = 0.002) and end-systolic (56 ± 21 vs 81 ± 45 mL/m², P = 0.007) volumes were significantly smaller in the *DSP* group as compared to the LV+ right-dominant-ACM group.

Both LVEDV/RVEDV [1.01 (IQR 0.81–1.18) vs 0.78 (IQR 0.68–0.85)] and LVESV/RVESV (0.96 [IQR 0.70–1.27] vs 0.59 [IQR 0.48–0.69] were significantly higher in the *DSP* group than in the LV+ right-dominant-ACM group (P < 0.0001 for both). The ROC AUC for discriminating the *DSP* status in the overall study population was 0.82 for LVEDV/RVEDV and 0.86 for LVESV/RVESV. The optimal cutoff for the latter was 0.8, with a sensitivity of 72% and a specificity of 91%.

#### 3.3.2 Dynamic and static ventricular morphological abnormalities

**Fig. 1:**
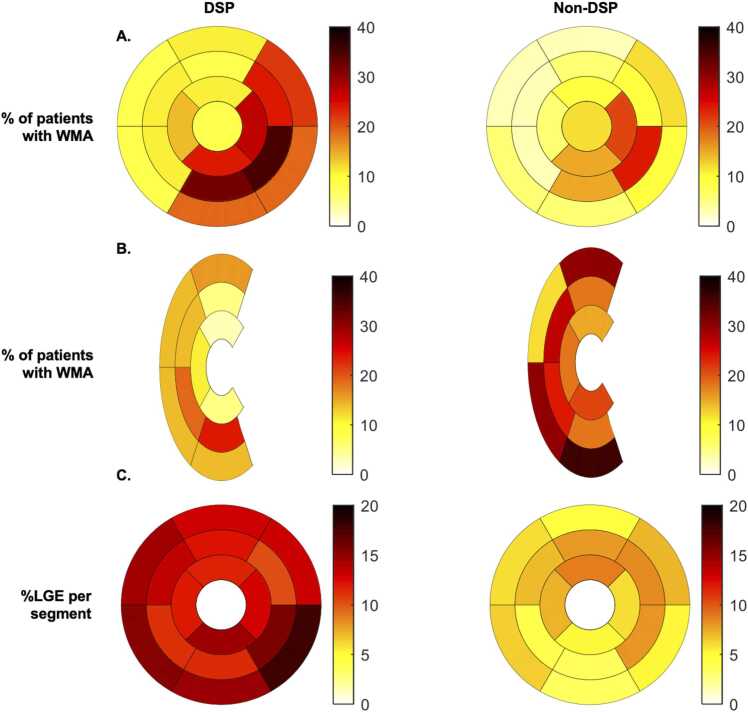
Distribution and frequency of any LV (A) and RV (B) wall motion abnormalities (WMA). The LV bulls-eye refers to the 17-segments AHA classification. The RV bulls-eye refers to a customized 12-segment segmentation excluding the septum (see Section 2). LV WMAs were more frequent in the *DSP*-ACM group, especially in the lateral and inferior walls, while RV WMAs were more frequent in the LV+ right-dominant ACM group. (C) %LGE per segments. Patients in the *DSP*-ACM group had an overall higher content of LV LGE, especially in the inferolateral wall. *ACM* arrhythmogenic cardiomyopathy, *AHA* American Heart Association, *DSP* desmoplakin, *LGE* late gadolinium enhancement, *LV* left ventricle, *RV* right ventricle.

Although a comparable number of patients had at least one LV WMA in both groups, the number of LV segments with WMA was higher in the *DSP*-ACM (2.5 [IQR 0–4]) than in the LV+ right-dominant-ACM group (0 [IQR 0–4], P = 0.04) (Table 3, Supplemental Table 1, and Fig. 1A). The LV lateral wall was the only LV wall in which patients in the *DSP*-ACM group had significantly more frequently a WMA in at least one segment (19/37 [51%] patients vs 10/33 [30%] patients, P = 0.04).

By contrast, although most patients overall had at least one RV WMA, fewer patients in the *DSP*-ACM group exhibited any RV WMA than in the LV+ right-dominant-ACM group (20/37 [54%] patients vs 26/33 [79%] patients, P = 0.04) (Fig. 1B). There was no significant difference in the distribution of static RV morphological abnormalities between both groups.

#### 3.3.3 Presence, extent, and patterns of left ventricular LGE

While a comparable number of patients had any LGE in both groups (Table 3), patients in the *DSP* group had significantly more LV segments with LGE (6.5 [IQR 1–14] vs 2 [IQR 1–6], P = 0.005) (Supplementary Table 2). Regionally, the most remarkable differences in LGE between the *DSP* and LV+ right-dominant-ACM groups were found in the LV septum (22/37 [61%] vs 6/33 [18%], P < 0.0001), the anterior wall (20/37 [54%] vs 6 [18%], P = 0.0004) and the inferior wall (24/37 [65%] vs 14 [42%], P = 0.0004); whereas LGE in the lateral wall was similar in both groups (26/37 [70%] patients in the *DSP* group vs 23/33 [70%] patients in the LV+ right-dominant-ACM group, P = 0.81). Additionally, among the 17 patients who had LGE in all 4 LV walls, 15/17 (88%) belonged to the *DSP* group (Supplementary Fig. 2).

Across both groups, a total of 372 (31%) LV segments had LGE. The LGE pattern was subepicardial in 346/372 (93%) segments, intramyocardial in 13/372 (3%) segments, transmural in 12/372 (3%) segments, and subendocardial in 1/372 (0.3%) segment. Among segments with subepicardial LGE, 260/346 (75%) had a transmural extent <50%. The LV segments with transmural extent ≥50% were predominantly the basal inferoseptal (31/70, 44%) and the basal inferior (27/70, 38%) segments. LGE transmural extent ≥50% was significantly more present in the *DSP* group (18/37 [50%] patients vs 7/33 [21%], P = 0.01). A ring pattern was found almost exclusively in the *DSP* group (16/37 [43%] patients vs 1/33 [3%], P < 0.0001), leading to a specificity of 97%.

**Fig. 2:**
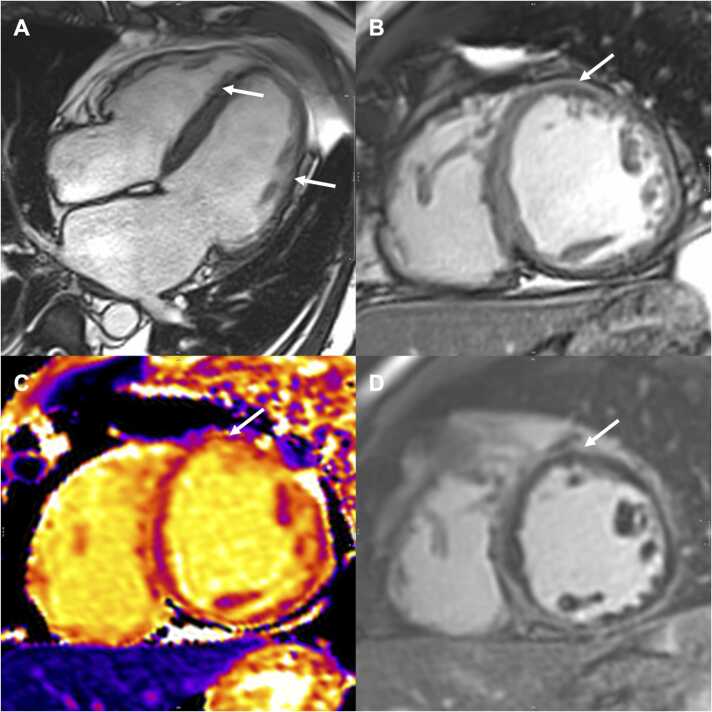
Four-chamber (A) and mid short-axis (B-D) SSFP images in a 79-year-old man with a *DSP* pathogenic variant and ACM with LV involvement. Four-chamber view showed LV dilatation (LVEDVi = 139 mL/m², LVESV/RVESV: 1.3 with lateral, inferior, and anteroseptal) LV thinning due to fatty infiltration (arrows). Cine sequence short-axis sequence performed after injection depicted an early gadolinium enhancement in the anteromedial segment (arrow), also seen in the short-axis native T1 with an elevation of the regional T1 relaxation time at 1251 ms (arrow). Short-axis LGE sequence showing circumferential LV subepicardial LGE involving all LV basal segments including the anterior wall (arrow) and referred to as a “ring sign.” *ACM* arrhythmogenic cardiomyopathy, *DSP* desmoplakin, *LGE* late gadolinium enhancement, *LV* left ventricle, *LVEDVi* indexed left ventricular end-diastolic volume, *LVESV* left ventricular end-systolic volume, *RVESV* right ventricular end-systolic volume, *SSFP* steady-state free precession.

**Fig. 3:**
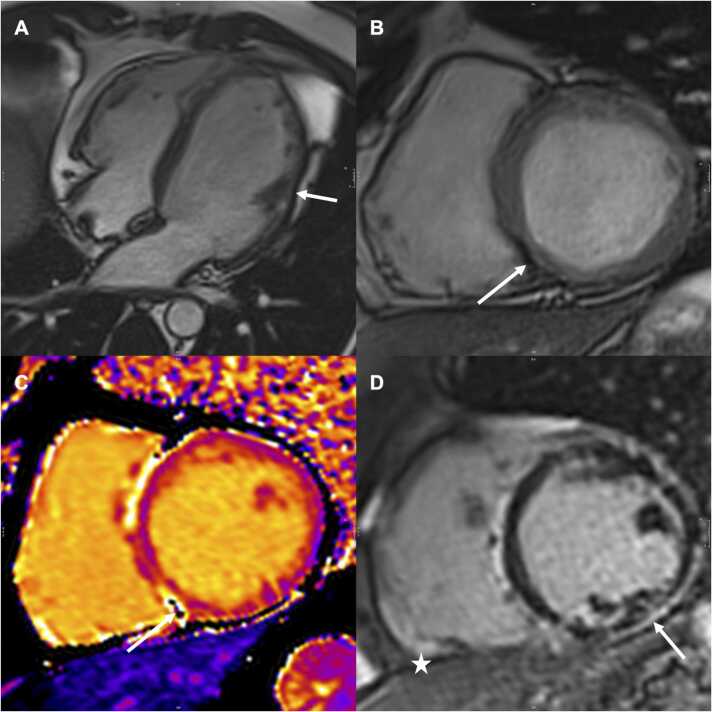
Four-chamber (A) and short-axis (B-D) images in a 43-year-old man with a *DSP* pathogenic variant and ACM with predominant LV involvement. Four-chamber view showing LV dilatation (LVEDVi = 116 mL/m², LVESV/RVESV: 1.1), lateral LV thinning, and fatty infiltration (arrow). Short-axis cine sequences revealed inferoseptal fatty infiltration (arrow), also seen in the short-axis native T1 with the corresponding hyposignal on the mapping reconstruction (arrow). Short-axis LGE sequence showing an LV subepicardial enhancement “stria” pattern involving the anteroseptal, inferoseptal, inferior, and inferolateral segments, referred to as a “ring sign.” *ACM* arrhythmogenic cardiomyopathy, *DSP* desmoplakin, *LGE* late gadolinium enhancement, *LV* left ventricle, *LVEDVi* indexed left ventricular end-diastolic volume, *LVESV* left ventricular end-systolic volume, *RVESV* right ventricular end-systolic volume.

**Fig. 4:**
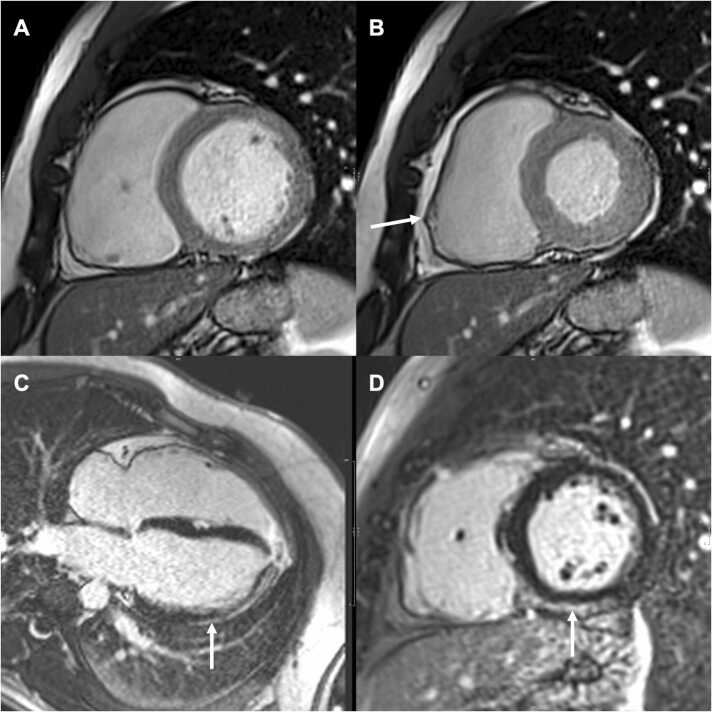
Short-axis (A, B, D) and four-chamber (C) bright blood images in a 50-year-old man with definite (2010 TFC) ARVC with a *PKP2* pathogenic variant and biventricular involvement. End-diastolic (A) and end-systolic(B) SA images showed an RV dilatation (RVEDVi = 136 mL/m²) and an inferolateral bulging and dyskinesia (arrow) of the right ventricle. Four-chamber view (C) and short-axis (E) LGE sequence highlighted LV subepicardial enhancement (arrows) sparing the anterior, anteroseptal, and inferolateral wall. Despite the presence of a subepicardial LGE “stria” pattern, a ring-like pattern was not reached as LGE did not involve ≥3 contiguous basal LV segments (≥50% of the LV circumference) in the same short-axis slice, and LVESV/RVESV was 0.7 (note <0.8). *ARVC* arrhythmogenic right ventricular cardiomyopathy, *LGE* late gadolinium enhancement, *LV* left ventricle, *LVESV* left ventricular end-systolic volume, *PKP2* plakophilin-2, *RV* right ventricle, *RVEDVi* indexed right ventricular end-diastolic volume, *TFC* Task Force Criteria.

Case examples of patients in the *DSP*-ACM and the LV+ right-dominant-ACM groups are shown in Fig. 2, Fig. 3, Fig. 4 .

#### 3.3.4 LGE quantification

Global LGE mass expressed as a percentage of LV mass (%LGE) was significantly higher in the *DSP* group as compared to the LV+ right-dominant-ACM group (14 ± 16 vs 2 ± 3%, P < 0.0001). The distribution of %LGE across LV segments is shown in Fig. 1C. Most LV segments had a higher %LGE in the *DSP* than in the LV+ right-dominant-ACM groups. The ROC AUC for %LGE to identify *DSP* was 0.76. A %LGE value >4% had a sensitivity of 68% and a specificity of 83% to differentiate *DSP*-ACM from LV+ right-dominant-ACM, and an %LGE value >5% was associated with a specificity of 95%. Of note, basal inferior and basal inferolateral segments showed maximal difference in %LGE between both groups. There was a strong correlation between quantitative %LGE and visually assessed number of LV segments with LGE in both the *DSP* (Spearman ρ = 0.90, P < 0.0001) and the LV+ right-dominant-ACM (Spearman ρ = 0.75, P < 0.0001) groups.

#### 3.3.5 Myocardial mapping

Although native myocardial T1 was slightly higher in the *DSP* than in the LV+ right-dominant-ACM group (1042 ± 58 vs 1017 ± 39 ms), the difference did not reach statistical significance. Myocardial T2 was not different between groups.

### 3.4 Clinical outcomes

Among 59 patients with follow-up data available, 13/60 (22%) patients experienced sustained VA during a median follow-up of 1.5 years: 6/37 (16%) in the *DSP*-ACM group and 7/23 (30%) in the LV+ right-dominant ACM group (P = 0.10). Four patients experienced ≥1 episode of congestive HF: 3 in the *DSP*-ACM group and 1 in the LV+ right-dominant ACM group. There was no death, and one patient in each group underwent heart transplantation. Eight patients presented the composite outcome in the *DSP* group and seven patients in the LV+ right-dominant ACM group.

In the *DSP*-ACM group, LVEF and RVEF were significantly lower in patients who presented the composite outcome (38% (IQR 27–48) and 37% (IQR 28–44), respectively) than in patients who did not [50% (IQR 42–57], P = 0.01% and 49% [IQR 43–56%], P = 0.005). There was no significant difference in LVESV/RVESV in patients who presented the composite outcome (1.06 [IQR 0.68–1.36]) than in patients who did not (0.93 [IQR 0.70–1,22], P = 0.56). Despite a trend, there was no significant difference in the amount of LV LGE in patients who presented the composite outcome (22% [IQR 4–43%]) than in patients who did not (7% [IQR 1–13%], P = 0.11).

LVEF (odds ratio [OR] for a 1%-increase 0.91, 95% confidence interval [CI] 0.84–0.99, P = 0.03) and RVEF (OR for a 1%-increase 0.90, 95% CI 0.83–0.98, P = 0.01) were associated with a reduced occurrence of the composite outcome. The amount of LGE was also associated with the composite outcome (OR for a 1%-increase 1.05, 95% CI 1.00–1.10, P = 0.04).

## 4 Discussion

The novelty of this study is twofold, including a comprehensive characterization of CMR features in *DSP*-ACM with a head-to-head comparison of these features to those obtained in patients with ACM related to non-*DSP* desmosomal genes with LV involvement.

Our main findings are (1) *DSP*-ACM was associated with significantly reduced LVEF and global 3D LV strains as compared to LV+ right-dominant-ACM; (2) LV WMA were more frequent in *DSP*-ACM patients and independent of the severity of RV dilation/dysfunction; (3) the LV-to-RV volume ratio was the most potent volumetric discriminator between *DSP*-ACM and LV+ right-dominant-ACM patients, with an AUC of 0.86; (4) patients in the *DSP*-ACM group had more LV segments with LGE, and the extent of LGE was significantly higher in *DSP*-ACM patients, particularly in the lateral and inferior walls. Global LGE burden measured as %LGE >5% was highly specific of *DSP*-ACM; (5) the presence of a ring-like pattern corresponding to circumferential subepicardial LGE involving more than three LV basal contiguous segments was highly specific of *DSP*-ACM.

### 4.1 LV involvement in ACM

ACM represents a collection of inherited cardiac disorders associated with VAs which may result in sudden cardiac death and HF [23]. Originally described as ARVC, characterized primarily by RV abnormalities [24], it has been acknowledged since the early 2000s that LV involvement is significant for both diagnosis and prognosis [12], [25]. In certain ACM variants, LV abnormalities may even predominate [5], [7], [26]. Consequently, the term ACM is now widely adopted to encompass the biventricular nature of the condition, while ARVC is retained to describe the traditional RV-dominant presentation [7], [27].

Pathological and autopsy reports initially identified LV involvement in ARVC [28], revealing fibrofatty replacement of LV myocardium in 76% of ARVC patients who succumbed to sudden cardiac death, raising questions about its prognostic significance. The RV, LV fibrofatty remodeling progresses from the epicardial to the endocardial layers, is frequently restricted to the former [12], [13], [14], [29], [30]. CMR tissue characterization plays a pivotal role in assessing LV involvement, as subepicardial fibrosis may only minimally affect LV systolic function particularly when measured by ejection fraction [13], [14]. Early cohort studies reported LV involvement in 7–24% of cases, whereas recent research indicates a notably higher prevalence, between 47% and 60%, suggesting that increased recognition of LV involvement and evolving CMR techniques have led to increased detection rates [13], [14], [25], [29], [30], [31], [32], [33]. Advances in genetic characterization of cardiomyopathies have identified some desmosomal genes such as *DSP* and non-desmosomal genes such as *Phospholamban*, *Filamin-C*, and *Transmembrane Protein 43*, that are often associated with biventricular or LV-dominant subforms.

The prevalence of LV WMAs and systolic dysfunction has considerable variability. LV WMA predominate in the lateral and posterolateral LV segments [12], [13], [14], [29], [30], [31], [34]. Global LV systolic impairment is rare and associated with diffuse and frequently circumferential LV LGE. In contrast to DCM, LV LGE is the hallmark of LV involvement related to ACM and is frequently observed without corresponding WMA. Several studies of various size have found an LV LGE prevalence ranging between 0% and 68% [12], [13], [14], [29], [30], [34], [35]. LV LGE usually appears as a “band” in the epicardial and/or intramyocardial layers, sparing the endocardium and often affecting multiple segments. LGE primarily involves the lateral and inferolateral walls, forming the “displaced triangle of dysplasia.” This differs from the original “triangle of dysplasia,” which included the RV apex, now considered as a feature of advanced RV disease [32]. In some patients, septal and anterior LGE is observed, expanding lateral LV LGE toward a circumferential “ring-like pattern” [11], [13], [36], [37], [38].

Overall, LV involvement was once viewed as an end-stage ARVC feature, with LGE presumed to spread from the inferior RV to the LV through the inferoseptal region. This concept is now challenged, first and foremost through the recognition of LV-dominant ACM subforms involving DSP or non-desmosomal variants, as this article further explores, but also because studies report LV LGE in up to 43% and LV dysfunction in 16% of PKP2 variant carriers—the genotype most associated with “classical” ARVC—even without severe RV disease.

### 4.2 Prevalence, extent, and patterns of LGE in *DSP*-ACM

Since its original description, *DSP*-ACM is known as a highly fibrotic disease, and several histopathological reports and series have consistently shown circumferential subepicardial LV fibrofatty remodeling, with a variable predominance of the fatty and fibrotic components [2], [4], [39], [40], [41]. In parallel to these findings, cohorts of patients with *DSP*-ACM including CMR characterizations have reported a high but variable prevalence of LV LGE, ranging from 40 to 100% [5], [8], [9], [13], [14], [42]. Among 107 patients with Class IV/V *DSP* variants, Smith et al. reported a 40% prevalence of LV LGE in 57 patients who underwent CMR [5]. The prevalence of LV LGE was comparable in another study also including 91 Class IV/V *DSP* patients. Bariani et al. have reported a 70% LGE prevalence [9]. These variations may result from the systematic or elective enrollment of genotype+/phenotype− family members. In a cohort of patients with ACM, mixed genotypes and a majority of patients with LV involvement, all of the 19 *DSP*-ACM patients had LV LGE [14]. Several reports indicate the presence of isolated LV LGE in Class IV/V variants carriers otherwise asymptomatic and without any other phenotypical expression, suggesting that LV fibrosis occurs early in the course of the disease [5], [8], [12]. In line with the literature, we report an 86% prevalence of LV LGE in our *DSP*-ACM patients. Subepicardial LGE stands as the predominant LGE pattern in ALVC and has been consequently reported as such in ALVC and *DSP* cohort studies [13], [37], [42]. Consistently, in our study, subepicardial LGE represented 85% of LGE patterns in the *DSP*-ACM subgroup. Despite the presence of LGE, LV function was overall only mildly altered, and the correlation between LVEF or LV global longitudinal strain and LGE burden was poor. This may result from regional LGE heterogeneity in less severe cases with spared segments able to functionally compensate for segments with LGE. However, this is probably also related to the fact that volumetric changes required for LVEF measurement are based on endocardial segmentation and that epicardial damage has relatively less functional consequences on global myocardial deformation than endocardial damage because of the decreasing endo to epicardial motion gradient. Indeed, several *DSP*-ACM patients exhibited normal LV deformation parameters despite diffuse epicardial LGE. This finding is probably related to the lesser impact of the epicardial layer on LV contractility.

Lateral and inferolateral LV LGE is now established as a typical feature of all subtypes of ACM. In “classic” ARVC, LV LGE, mostly inferolateral, is seen in up to 35–68% of patients and while often associated with advanced RV disease, it is also prevalent in patients with mild disease expression [25], [31], [33]. In *DSP*-ACM as in other genotypes associated with ALVC, the inferior and inferior lateral LV wall is also the region harboring most LGE [5], [8], [9]. Smith et al. reported a constant presence of inferior LV LGE in a subset of 10 *DSP* patients who had CMR [5]. In a genotype-imaging correlational study including a cluster of *DSP* carriers, Augusto et al. found a 69% LGE presence in the subepicardial inferolateral LV but that study also included patients with filamin-C Class IV/V variants [13]. Our findings are consistent, but we found that both *DSP* and LV+ right-dominant-ACM had inferior and lateral LGE in a comparable proportion. However, *DSP*-ACM patients frequently had anterior and septal LGE, a finding that was very rare in LV+ right-dominant-ACM. Considering that age at CMR was similar in both groups, this may suggest an early and more severe development of epicardial fibrosis in *DSP* compared to LV+ right-dominant-ACM patients.

The ring-like pattern, described since the initial reports of LV-dominant ACM by Sen-Chowdhry et al., is generally referred to as “stria” subepicardial pattern affecting more than two contiguous LV segments within the same LV short-axis slice [13], [37]. Using this definition, the feature was present in a significant proportion of patients with LV+ right-dominant-ACM in the present study. However, when the ring-like pattern was defined as the presence of LGE in more than 50% of the LV circumference (≥3 consecutive segments), it became a hallmark of *DSP*-ACM. Nevertheless, the fully circumferential LGE ring was present in only a minority of patients with a ring-like pattern.

### 4.3 The clinical relevance of a comprehensive evaluation of morphological abnormalities in *DSP*-ACM

In “classic” ARVC, RV dysfunction has been linked to VA, sudden death, and HF in multiple studies, since the early days of the disease recognition [6], [43]. However, the impact of LV dysfunction on prognosis has been debated. In several studies based on angiography, echocardiography, or CMR, LVEF was found to be an independent predictor of VAs and appropriate discharge by implantable defibrillators [25], [44], [45], [46]. In contrast, in the larger prospective multicenter study including ARVC patients with a definite TFC diagnosis and without previous VA, LVEF was not independently associated with VA occurrence during follow-up [47]. This discordance might be explained by the low prevalence of patients with LV involvement in cohorts, including patients on the basis of the 2010 TFC ARVC criteria, subsequently favoring the “classic” ARVC phenotype. As well, it may reflect the limitations of LVEF to detect underlying LV involvement, which may precede LVEF alteration. In that regard, our data suggest a potential value of deformation alterations measured as 3D strains in CMR to characterize early LV involvement.

*DSP*-ACM has been associated with variable phenotypes ranging from ARVC to DCM or, biventricular ACM and ALCV with predominant LV abnormalities [4], [5], [8], [9]. In the present study, RV regional and global morphological abnormalities remained prevalent in patients with *DSP*-ACM and were correlated with LV morphological abnormalities, a finding that confirms the mostly biventricular nature of myocardial involvement in *DSP*-ACM. Recently, a novel DSP-specific risk score derived from the international *DSP*-ERADOS cohort identified LV dysfunction, even mild (LVEF <50%) as a significant predictor of the arrhythmic risk. RV dysfunction was also a significant and independent predictor of worse outcomes, suggesting that both LV and RV alterations have a prognostic value in DSP-ACM [48], [49].

### 4.4 Insights from LV/RV interrelations

Several studies previously showed that *DSP*-ACM could embrace diverse morphological phenotypes relative to LV/RV predominant involvement [5], [8], [9]. In the study by Smith et al., up to 10% of *DSP*-ACM patients had a “classic” ARVC phenotype [5]. Our study shows several interesting aspects of the LV to RV interaction in *DSP*-ACM as compared to LV+ right-dominant-ACM. First, despite the inclusion of patients having LV involvement, most *DSP*-ACM patients had some degree of RV regional or global morphological abnormality and correlated degrees of LV and RV dysfunction suggesting, in line with the literature, that biventricular ACM is the most common phenotype associated with *DSP*-ACM [5]. Nevertheless, the LV-to-RV volume ratio was the most discriminative volumetric parameters between both groups, suggesting that LV involvement predominates or precedes in *DSP*-ACM, whereas RV involvement predominates in LV+ right-dominant-ACM. Also, in *DSP*-ACM, the degree of LV dysfunction, dilatation, and LGE extent were positively correlated. In contrast, LV dysfunction did not parallel dilatation and LGE extent in LV+ right-dominant-ACM, which may be due to overall less severe LV alterations but may also suggest that deleterious LV/RV interactions rather than primitive LV dysfunction related to fibrofatty replacement may be at play in the LV+ right-dominant-ACM subgroup.

### 4.5 Are *DSP*-ACM CMR features specific?

The fact that *DSP*-ACM has different CMR features than DCM and “classic” ARVC is already well established [6], [7], [13], [50]. In this study, we were able to identify several characteristics that distinguish *DSP*-ACM from LV+ right-dominant-ACM with LV involvement: (1) An LV-to-RV volume ratio >0.8, rather than both volumes taken independently; (2) the presence of epicardial anterior and septal LGE; (3) the presence of diffuse epicardial LGE with a ring-like pattern. Taken together, these features constitute a potential CMR signature that should lead to consider the diagnosis of *DSP*-ACM. However, whether this signature is also shared by other non-desmosomal genotypes previously associated with LV-dominant or biventricular ACM such as the rare PLN, *FLNC*, or *TMM43*, absent from our study, remains to be established. Available reports on these entities suggest that their morphofunctional and structural profile may be similar to that of *DSP*-ACM, including the ring-like pattern [51], [52].

## 5 Limitations

Our study has several limitations. First, its retrospective nature and the rare disease population subtypes studied led to CMR evaluations performed at various ages and stages of disease progression, hampering the study’s ability to provide reliable insights on the temporal relationships between CMR and clinical findings, which are both largely of an irreversible nature. Likewise, the varied timing of CMR evaluations and small sample size restrained any attempt to analyze the significance of CMR findings with regards to disease evolution and patient outcomes. Despite RV LGE being considered an important feature of ARVC described since the first ARVC CMR characterization studies, we believe that the thin-walled RV and the limited spatial resolution of two-dimensional LGE cannot be used to perform a reliable quantitative and regional analysis of RV LGE in numerous patients. Recently available high spatial resolution 3D LGE, now used in both institutions, was not performed in all patients; we consequently chose not to include RV LGE analysis to limit precision biases. Accordingly, we used the 2010 TFC ARVC criteria instead of the more recent 2020 IC Criteria, which include RV LGE as the main feature of the structural criterion. Nevertheless, the 2010 TFC criteria are still relevant for assessing the extent of RV disease and portraying the “classic” ARVC phenotype. Furthermore, myocardial mapping data were available for a limited number of patients in both groups, leading to small subsample sizes precluding their interpretation. Second, the LV+ right-dominant-ACM group comprised patients with P/LP variants in specific desmosomal genes, namely *PKP2* and *DSG2*, and no other non-desmosomal gene previously associated with LV-dominant or biventricular ACM. As a result, this group does not fully represent the entire spectrum of LV-dominant ACM. The clinical-CMR phenotype of these distinct entities has been previously described and much resembles that of *DSP*-ACM. Our aim was to compare features of *DSP*-ACM to the most commonly encountered genotypes when they are associated with LV involvement. Last, some patients had a *DSG2* pathogenic variant and a dual *PKP2* and *DSG2* P/LP variant. While there are conflicting data as to whether *DSG2* is associated with greater LV involvement as compared to *PKP2*-related ACM [34], this would not have accentuated the differences between the two groups.

Future prospective studies will help to decipher the natural history of morphofunctional and structural alterations in *DSP*-ACM, their relations with intercurrent events such as myocarditis episodes, and most importantly their prognostic significance in both quantitative and qualitative terms.

## 6 Conclusions

This study describes the CMR features found in *DSP*-related ACM compared to those found in LV+ right-dominant-ACM. Diffuse LV LGE extending beyond the inferolateral LV wall was the main structural alteration related to *DSP*-ACM. Among patients with ACM, an epicardial LGE ring-like pattern, involving ≥50% of the LV circumference was a distinctive feature of *DSP*-ACM. In a patient with ACM, the combined presence of diffuse LGE with an end-systolic LV-to-RV volume ratio ≥0.8 may be considered as indicative of a high pre-genetical test likelihood for *DSP*-ACM and therefore prompt careful risk assessment of lethal VAs associated with this disease.

## Funding source

None.

## Author contributions

**Alban Redheuil:** Writing – review & editing, Visualization, Validation, Supervision, Resources, Project administration, Methodology, Investigation, Formal analysis, Data curation, Conceptualization. **Etienne Charpentier:** Writing – original draft, Formal analysis. **Shannon Soulez:** Formal analysis, Data curation. **Nadjia Kachenoura:** Writing – review & editing, Visualization, Validation, Supervision, Software, Resources, Methodology, Formal analysis, Conceptualization. **Estelle Gandjbakhch:** Writing – review & editing, Visualization, Validation, Supervision, Resources, Project administration, Methodology, Investigation, Conceptualization. **Mikael Laredo:** Writing – review & editing, Writing – original draft, Methodology, Investigation, Formal analysis, Data curation, Conceptualization. **Annamaria Martino:** Data curation. **Leonardo Calò:** Data curation. **Vincent Nguyen:** Methodology, Formal analysis. **Véronique Fressart:** Methodology, Data curation. **Philippe Charron:** Data curation. **Flavie Ader:** Formal analysis. **Alexis Hermida:** Writing – review & editing, Formal analysis.

## Declaration of competing interests

The authors declare the following financial interests/personal relationships which may be considered as potential competing interests: Mikael Laredo reports a relationship with Boston Scientific Corporation that includes funding grants. Mikael Laredo reports a relationship with Boston Scientific Corporation that includes consulting or advisory. Mikael Laredo reports a relationship with Abbott Cardiovascular that includes consulting or advisory. Philippe Charron reports a relationship with Amicus Therapeutics Inc Research & Gene Therapy Center of Excellence that includes consulting or advisory. Philippe Charron reports a relationship with Bristol Myers Squibb Co. that includes consulting or advisory. Philippe Charron reports a relationship with Owkin France that includes consulting or advisory. Philippe Charron reports a relationship with Pfizer that includes consulting or advisory. Philippe Charron reports a relationship with Sanofi that includes consulting or advisory. Estelle Gandjbakhch reports a relationship with Biosense Webster Inc. that includes consulting or advisory. Estelle Gandjbakhch reports a relationship with Medtronic that includes consulting or advisory. Estelle Gandjbakhch reports a relationship with Abbott Cardiovascular that includes consulting or advisory. Estelle Gandjbakhch reports a relationship with Boston Scientific Corporation includes. Alban Redheuil reports a relationship with Imageens that includes equity or stocks. Alban Redheuil reports a relationship with Siemens Healthineers AG that includes travel reimbursement. The other authors declare that they have no known competing financial interests or personal relationships that could have appeared to influence the work reported in this paper.

## Footnotes

Appendix A Supplementary data associated with this article can be found in the online version at doi:10.1016/j.jocmr.2025.101867.
