# Supplementary material for: Imaging features of desmoplakin arrhythmogenic cardiomyopathy: A comparative cardiovascular magnetic resonance study
Source: J Cardiovasc Magn Reson. 2025 Feb 26;27(1):101867. doi: 10.1016/j.jocmr.2025.101867 (PMC12138554; doi:10.1016/j.jocmr.2025.101867)
Supplement: Supplementary file 1 — Supplementary material [file mmc1.docx]

**Supplementary Material**

**Supplementary Figures**

**Supplementary Figure 1.
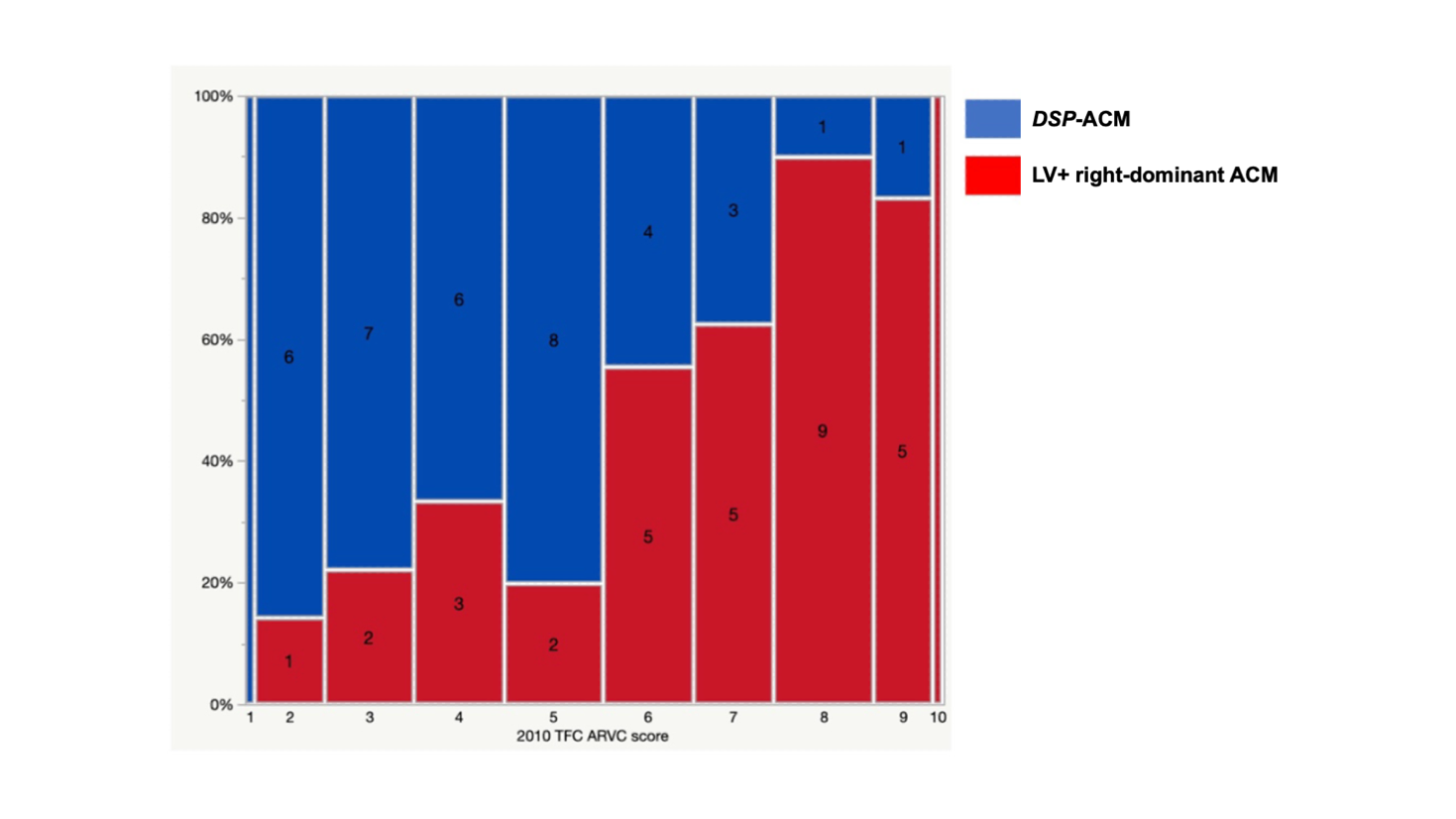
**

Distribution of DSP-ACM vs. LV+ right-dominant ACM patients relative to their 2010 TFC ARVC score

**Supplementary Figure 2.
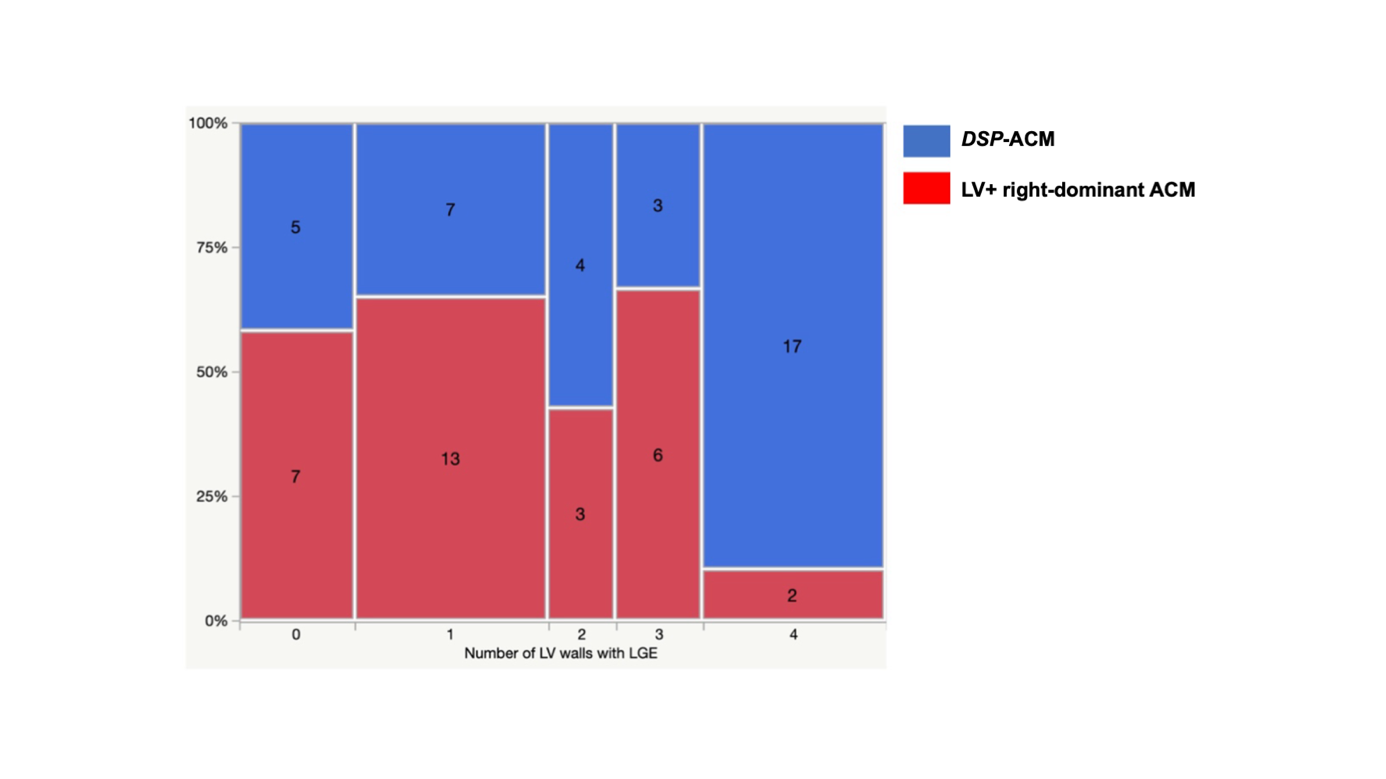
**

Distribution of DSP-ACM vs. LV+ right-dominant ACM relative to the number of LV walls (0—4) with LGE

**Supplementary Tables**

**Supplementary Table 1:** Biventricular dynamic and static morphological abnormalities

|  | **All patients (n=70)** | ***DSP* (n=37)** | **LV+ right-dominant ACM (n=33)** | **P-value** |
| --- | --- | --- | --- | --- |
| Left ventricle |  |  |  |  |
| ≥1 segment with WMA | 37 (53) | 22 (59) | 15 (45) | 0.2 |
| N° of segments with WMA | 1 (0—4) | 2.5 (0—4) | 0 (0—2) | 0.04 |
| ≥1 segment with akinesia | 8 (12) | 5 (14) | 3 (9) | 0.53 |
| N° of segments with akinesia | 0 (0—0) | 0 (0—0) | 0 (0—0) | 0.57 |
| ≥1 segment with dyskinesia | 4 (6) | 2 (6) | 2 (6) | 0.93 |
| N° of segments with dyskinesia | 0 (0—0) | 0 (0—0) | 0 (0—0) | 0.94 |
| ≥1 segment with WMA, anterior wall | 12 (17) | 8 (22) | 4 (12) | 0.26 |
| N° of segments with WMA, anterior wall | 0 (0—0) | 0 (0—0) | 0 (0—0) | 0.28 |
| ≥1 segment with WMA, lateral wall | 29 (42) | 19 (53) | 10 (30) | 0.04 |
| N° of segments with WMA, lateral wall | 0 (0—2) | 1 (0—2) | 0 (0—1) | 0.07 |
| ≥1 segment with WMA, inferior wall | 22 (31) | 15 (42) | 7 (21) | 0.06 |
| N° of segments with WMA, inferior wall | 0 (0—1) | 0 (0—1.75) | 0 (0—0) | 0.07 |
| ≥1 segment with WMA, septum | 11 (16) | 8 (22) | 3 (9) | 0.13 |
| N° of segments with WMA, septum | 0 (0—0) | 0 (0—0) | 0 (0—0) | 0.14 |
| Apical WMA | 7 (10) | 3 (8) | 4 (12) | 0.17 |
| Right ventricle |  |  |  |  |
| ≥1 segment with WMA | 20 (56) | 20 (56) | 26 (79) | 0.04 |
| N° of segments with WMA | 1 (0—3) | 1 (0—2) | 2 (1—4) | 0.04 |
| ≥1 segment with dyskinesia/asynchronism | 45 (65) | 19 (53) | 26 (79) | 0.02 |
| N° of segments with dyskinesia/asynchronism | 1 (0—2) | 1 (0—2) | 1 (1—3.5) | 0.07 |
| ≥1 segment with scalloping | 23 (33) | 10 (27) | 13 (39) | 0.27 |
| N° of segments with scalloping | 0 (0—1) | 0 (0—1) | 0 (0—2.5) | 0.23 |
| ≥1 segment with bulging | 16 (23) | 6 (16) | 10 (30) | 0.16 |
| N° of segments with bulging | 0 (0—0) | 0 (0—0) | 0 (0—1) | 0.15 |
| ≥1 segment with aneurysm | 13 (19) | 5 (14) | 8 (24) | 0.25 |
| N° of segments with aneurysm | 0 (0—0) | 0 (0—0) | 0 (0—0.5) | 0.32 |

Data are n (%) or median (interquartile range). Abbreviations. WMA: wall motion abnormality.

**Supplementary Table 2:** Left ventricular gadolinium enhancement and mapping

|  | **All patients (n=70)** | ***DSP* (n=37)** | **LV+ right-dominant ACM (n=33)** | **P-value** |
| --- | --- | --- | --- | --- |
| LV LGE |  |  |  |  |
| ≥1 LV segment with LGE | 57 (83) | 31 (86) | 26 (79) | 0.42 |
| N° of LV segments with LGE | 4 (1—10) | 6.5 (1—14) | 2 (1—6) | 0.005 |
| LGE distribution across LV segments |  |  |  |  |
| ≥1 segment with LGE, LV anterior wall | 26 (38) | 20 (56) | 6 (18) | 0.001 |
| N° of segments with LGE, LV anterior wall | 0 (0—2) | 1 (0—3) | 0 (0—0) | 0.0004 |
| ≥1 segment with LGE, LV lateral wall | 49 (71) | 26 (72) | 23 (70) | 0.81 |
| N° of segments with LGE, LV lateral wall | 2 (0—5) | 3 (0—5) | 1 (0—3) | 0.11 |
| ≥1 segment with LGE, LV inferior wall | 31 (55) | 24 (67) | 14 (42) | 0.04 |
| N° of segments with LGE, LV inferior wall | 1 (0—2) | 2 (0—3) | 0 (0—1) | 0.003 |
| ≥1 segment with LGE, LV septum | 28 (41) | 22 (61) | 6 (18) | 0.0006 |
| N° of segments with LGE, LV septum | 0 (0—2) | 1 (0—3) | 0 (0—0) | <0.0001 |
| Any apical LGE | 7 (10) | 3 (8) | 4 (12) | 0.60 |
| LV LGE pattern |  |  |  |  |
| ≥1 segment with subepicardial LGE | 55 (80) | 31 (86) | 24 (72) | 0.17 |
| N° of LV segments with subepicardial LGE | 2 (1—10) | 6 (1—13) | 2 (0—5) | 0.003 |
| ≥1 segment with ≥50% subepicardial LGE | 25 (36) | 18 (50) | 7 (21) | 0.01 |
| N° of LV segments with ≥50% subepicardial LGE | 0 (0—15) | 0.5 (0—3) | 0 (0—0) | 0.02 |
| ≥1 segment with subendocardial LGE | 1 (1) | 1 (1) | 0 | 0.25 |
| N° of LV segments with subendocardial LGE | 0 (0—0) | 0 (0—0) | 0 (0—0) | 0.35 |
| ≥1 segment with intramyocardial LGE | 10 (14) | 5 (14) | 5 (15) | 0.88 |
| N° of LV segments with intramyocardial LGE | 0 (0—0) | 0 (0—0) | 0 (0—0) | 0.92 |
| ≥1 segment with transmural LGE | 5 (7) | 2 (6) | 3 (9) | 0.57 |
| N° of LV segments with transmural LGE | 0 (0—0) | 0 (0—0) | 0 (0—0) | 0.60 |
| Ring-like pattern^a^ | 17 (24) | 16 (43) | 1 (3) | <0.0001 |

Data are n (%), mean ± SD or median (interquartile range). a: defined as a “stria” subepicardial LGE involving ≥3 contiguous basal LV segments (≥50% of the LV circumference) in the same short-axis slice. Abbreviations. LGE: late gadolinium enhancement; LV: left ventricle
